# Hyperthermic intrathoracic chemotherapy in the management of pleural malignancies: current evidence, limitations, and future directions

**DOI:** 10.36416/1806-3756/e20250504

**Published:** 2026-03-05

**Authors:** Pedro Henrique Xavier Nabuco de Araujo, Fabio Eiti Nishibe Minamoto, Paulo Manuel Pêgo-Fernandes

**Affiliations:** 1. Hospital das Clínicas - HCFMUSP - Faculdade de Medicina, Universidade de São Paulo, São Paulo (SP) Brasil.; 2. Disciplina de Cirurgia Torácica, Faculdade de Medicina da Universidade de São Paulo - FMUSP - São Paulo (SP) Brasil.; 3. Departamento de Cardiopneumologia, Faculdade de Medicina da Universidade de São Paulo - FMUSP - São Paulo (SP) Brasil.; 4. Departamento Científico, Associação Paulista de Medicina - APM - São Paulo (SP) Brasil.

Hyperthermic intrathoracic chemotherapy (HITHOC) has gained increasing relevance in the management of pleural malignancies, particularly malignant pleural mesothelioma (MPM) and stage IVa thymoma. Although the technique has been incorporated heterogeneously across specialized centers, the available evidence suggests that its role as an adjunct to cytoreductive surgery requires further investigation. The present discussion, supported by large retrospective datasets and institutional series, aims to outline the current state of knowledge, persistent gaps, and requirements for long-term consolidation of the technique.

The concept of hyperthermic intra-cavity chemotherapy was first introduced for the peritoneal cavity as hyperthermic intraperitoneal chemotherapy (HIPEC), aimed at treating metastatic abdominal tumors. It was designed as an adjuvant therapy to extensive cytoreductive surgery, with the goal of improving patient survival. This technique was initially described by Spratt in 1980 as a thermal transfusion infiltration system, tested on canine models, to manage malignant effusions from metastatic abdominal cancers.^1^ Since then, HIPEC has gained prominence and become an integral component of multimodal strategies for managing both primary and secondary peritoneal tumors. 

Intrathoracic chemotherapy was first described in 1955 by Bateman et al.,^2^ who reported the intracavitary instillation of triethylene thiophosphoramide into the pleural, peritoneal, or pericardial spaces following therapeutic drainage in ambulatory patients. A key contribution of this early work was the demonstration that recurrent malignant pleural effusions responded particularly well to local chemotherapy, with more durable control than that observed for malignant ascites. The authors emphasized that intracavitary administration allowed drug concentrations substantially higher than those achievable systemically, leading to superior local tumor control and even ancillary effects on distant disease. Importantly, they reported no early clinical toxicity and no histological injury to nontumoral tissues, supporting the safety and feasibility of this strategy for managing recurrent malignant pleural effusion in the outpatient setting.^2^

Recent review articles have synthesized evidence from multiple series and consistently report wide variability in median survival, yet suggest a modest but meaningful incremental benefit when HITHOC is combined with macroscopic complete resection.^3^ ^,^ ^4^ The effect appears particularly pronounced in patients with epithelioid histology, in whom microscopic residual disease is more responsive to locoregional cytotoxic exposure. Similarly, studies focused on thymoma demonstrated remarkably stable 5-year survival rates ranging from 70% to 90% among patients with stage IVa disease or pleural recurrence treated with pleural metastasectomy followed by HITHOC.^5^ ^,^ ^6^ Despite the inherent limitations of small sample sizes and single-center designs, these findings have been reproduced across European and Asian cohorts, reinforcing the biological plausibility and clinical consistency of this approach.

The largest retrospective analysis to date, encompassing more than 3,200 patients with MPM undergoing surgical resection, demonstrated that the addition of HITHOC was associated with improved overall survival in a multivariable model (HR = 0.80; 95% CI: 0.69-0.92; p = 0.002). This survival advantage persisted after propensity-score matching (HR = 0.73; 95% CI: 0.61-0.88; p = 0.001).^7^ Although patients receiving HITHOC experienced longer hospital stays (12 vs. 7 days; p < 0.001) and higher 30-day readmission rates (9.9% vs. 4.9%; p = 0.007), they demonstrated lower 30-day mortality (3.2% vs. 6.0%; p = 0.017) and lower 90-day mortality (7.5% vs. 10.9%; p = 0.0002), indicating that the technique introduces manageable morbidity without compromising-and potentially improving-perioperative safety.

Preclinical evidence provides a clear biological rationale for the use of HITHOC. Experimental studies^8^ ^-^ ^11^ demonstrated that combining cisplatin with hyperthermia at 41-43 °C increases drug penetration into pleural tissues, promotes apoptosis through oxidative and mitochondrial pathways, and enhances cellular membrane permeability. Penetration depth may reach 3.0-7.5 mm-particularly after decortication-consistent with the therapeutic objective of eliminating microscopic residual disease not amenable to further resection. Systemic absorption remains low, with serum cisplatin levels typically below 2% of the administered dose,^4^ thereby reducing the risk of nephrotoxicity and myelosuppression.

Despite the strong biological rationale and encouraging clinical outcomes, broader adoption of HITHOC has been limited by the lack of standardized protocols. Several authors have described marked variation across centers in essential technical aspects of the procedure, including drug regimen (cisplatin alone vs. combinations), dose (50-225 mg/m²), perfusion duration (60-120 minutes), target temperature, perfusate volume, and circuit configuration.^3^ ^-^ ^5^ ^,^ ^12^ This variability reduces comparability between studies, weakens the conclusions of meta-analyses, and slows incorporation into international guidelines. This challenge is reflected in recent ERS/EACTS/ESTS statements, which avoid providing specific procedural recommendations due to inconsistent reporting across the literature.

Efforts toward standardization, such as the German multicenter consensus,^3^ represent meaningful progress. The proposal of minimum technical parameters-including cisplatin doses between 150 and 175 mg/m², a target temperature of 42°C, and 60-min perfusion-offers a practical framework to harmonize protocols and improve reproducibility. In Brazil, recent publications have described robotic cytoreductive surgery followed by HITHOC,^13^ expanding the discussion on integrating minimally invasive approaches. Robotic platforms may provide improved visualization of the thoracic cavity and greater precision during cytoreduction, supporting their potential role in standardized treatment workflows.

Evidence outside MPM and thymoma remains limited. In non-small cell lung cancer with pleural dissemination, small institutional series summarized by Danuzzo et al.^4^ report median survival times between 15 and 33 months after cytoreduction plus HITHOC. However, heterogeneity in disease burden and surgical approach precludes definitive conclusions. In malignant pleural effusion from breast or ovarian cancer, the literature remains sparse, outdated, and methodologically inconsistent, restricting clinical applicability.

Thus, the central question is no longer whether HITHOC has potential utility, but how to advance toward its systematic consolidation. Three themes emerge consistently across the literature: 1- the technique is feasible and safe when performed in specialized centers; 2- there is a consistent signal of benefit in overall survival and locoregional control, particularly in MPM and thymoma; 3- methodological heterogeneity remains the primary barrier to broader adoption.

Future progress will require prospectively designed multicenter studies with standardized protocols, clearly defined eligibility criteria, and incorporation of functional and quality-of-life outcomes. The development of a pleural cancer burden index-analogous to the Peritoneal Cancer Index used in HIPEC-has been proposed^13^ as a strategy to enhance prognostic stratification and comparability across studies. 

The challenge ahead lies not in the lack of data, but in the need to organize and harmonize existing evidence. HITHOC currently occupies an intermediate position between promising locoregional therapy and formal guideline adoption. Consolidating its role in thoracic oncology will depend on methodological rigor, protocol standardization, and coordinated collaboration across centers-essential elements for transforming a technique with substantial potential into an established component of multimodal treatment strategies.
